# Swift mobilization of infection control, employee health, clinicians, engineering, laboratory and public health averted secondary cases following a large measles exposure at the British Columbia Children’s Hospital, Vancouver, BC, Canada

**DOI:** 10.1186/1753-6561-5-S6-O79

**Published:** 2011-06-29

**Authors:** E Thomas, S Dobson, G Al-Rawahi, L Holmes, R Gustafson, S Papilla, L Hoang, P Tilley

**Affiliations:** 1 B.C. Children's Hospital, Vancouver, BC, Canada; 2 Vancouver Coastal Public Health, Vancouver, BC, Canada; 3 Provincial Health Services Authority, Vancouver, BC, Canada

## Introduction / objectives

During a measles outbreak in British Columbia in 2010, a patient with clinical measles, confirmed by a positive IgM on the same day, was admitted to the Children’s Hospital. The patient's room was later determined not to meet airborne isolation standards, leading to a series of patient and staff exposures over 24 hours.

## Methods

A massive collaborative effort ensued, involving numerous hospital departments, to identify/ensure control of potentially infected individuals.

## Results

*Patients:* 52 patients were exposed: 16, who were immunocompromised, received prophylactic Immune Globulin, 21 had received 2 MMR doses, 3 had 1 dose, 6 were seropositive and 6 patients’ immunity remained unknown. *Staff:* 221 staff members were exposed; 103 were immune, 118 had unknown immunity [17 produced immunization record; 22 remained unknown; 79 were tested (71/79 seropositive, 6 susceptible, 2 “equivocal”)]. 21 staff members were furloughed from work, representing 231 worker-days. None of those exposed developed measles.

## Conclusion

This exposure illustrates the importance of early clinical recognition, rapid laboratory testing, measles risk awareness and strict adherence to airborne isolation protocols. An ongoing education program was created to ensure proper clinical management and infection control measures for all patients with rashes. A tight maintenance program for ventilation monitoring and a database for managers to access staff immunity records were also implemented.

## Disclosure of interest

None declared.

